# Chinese Herbal Medicine for Aspirin Resistance: A Systematic Review and Meta-Analysis

**DOI:** 10.1371/journal.pone.0154897

**Published:** 2016-05-06

**Authors:** Hanyu Chen, Zhengjie Shen, Jiandong Chen, Haowen Zhang, Xiaohu Chen

**Affiliations:** 1 First Clinical Medical College, Nanjing University of Chinese Medicine, Nanjing, China; 2 Department of Cardiology, Jiangsu Province Hospital of Traditional Chinese Medicine, the Affiliated Hospital of Nanjing University of Chinese Medicine, Nanjing, China; 3 Translational Medicine Center of Nanjing University of Chinese Medicine, Key Laboratory of Famous Doctor's Proved Recipe Evaluation and Transformation of State Administration of Traditional Chinese Medicine, Nanjing, China; Shanghai Jiao Tong University School of Medicine, CHINA

## Abstract

**Objectives:**

To assess the effectiveness and safety of Chinese herbal medicine (CHM) for the treatment of aspirin resistance (AR).

**Methods:**

A comprehensive research of seven electronic databases was performed for comparative studies evaluating CHM for AR. Two authors independently extracted data and assessed the methodological quality of the included trials using the Cochrane risk of bias tool. Data wasere synthesized by using RevMan 5.3 software. (PROSPERO Registration #CRD42015020182)

**Results:**

18 randomized controlled trials (RCTs) involving 1,460 patients were included. 15 RCTs reported significant difference in the reduction of platelet aggregation rate (PAR) induced by adenosine diphosphate (ADP) (P<0.05), and 11 reported significant effect of CHM plus aspirin to reduce PAR induced by arachidonic acid (AA) (P<0.05) compared with aspirin 100mg/d treatment. The pooling data of 3 RCTs showed the thromboxane B_2_ (TXB_2_) in patients with CHM plus aspirin versus aspirin were significantly reduced (Random Effect model (RE), Standard Deviation (SD) = -95.93, 95% Confidential Interval (CI)[-118.25,-73.61], P<0.00001). Subgroup analysis showed that TXB_2_ (Fixed Effect model (FE), SD = -89.23, 95%CI[-121.96,-56.49], P<0.00001) had significant difference in Tongxinluo capsule plus aspirin versus aspirin. 2 RCTs reported the clinical effective rate, and the meta-analysis result showed a significant difference in intervention and control group (FE, Relative Risk (RR) = 1.67, 95%CI[1.15, 2.42], P = 0.007<0.05). In 4 trials, CHM plus aspirin had better effects of reducing the reoccurrence of cerebral infarction than aspirin (FE, RR = 0.24, 95%CI [0.11, 0.49], P<0.0001). And one trial showed that CHM plus aspirin could decrease the National Institutes of Health Stroke Scale (NHISS) score (P<0.05) and increase the Barthel Index (BI) score (P<0.05). 4 trials stated that there were no adverse effects occurred in intervention group, and analysis showed significant difference of CHM or CHM plus aspirin in reducing the occurrence of adverse events (FE, RR = 0.22, 95%CI[0.13, 0.39], P<0.00001). 5 trials claimed that the CHM monotherapy and CHM adjunctive therapy for AR did not add the risk of bleeding (FE, RR = 0.50, 95%CI[0.20, 1.22], P = 0.13>0.05).

**Conclusions:**

CHM may be effective and safe as an alternative and collaborative therapy for AR. However, the current evidence and potential promising findings should be interpreted with caution due to poor and varying methodological quality of included studies and the heterogeneity of interventions. Thus, further exploration of this strategy with adequately powered RCTs is warranted.

## Introduction

Aspirin resistance (AR) is the incapacity of aspirin to decrease platelet production of thromboxane (TX) A_2_ and therefore platelets activate and aggregate [1]. That is to say, most aspirin-treated people still retain at substantial risk of clinically important cardiovascular events (CVE) due to insufficient inhibition of platelets, especially via the TXA_2_ pathway. AR can be divided into two types: clinical resistance and laboratorial resistance [2].

Despite the effective clinical efficacy and safety of aspirin for primary and secondary prevention of cardiovascular disease, new adverse cardiac events in aspirin-treated patients have been observed. It is reported that long-term aspirin-treated patients who are resistant to aspirin are at a greater risk of important cardiac and thrombotic morbidity than patients who are sensitive to aspirin [3]. The prevalence rate of AR has been estimated between 5% and 60% of aspirin-treated patients for secondary prevention [1,4]. And Chinese and overseas epidemiological investigations in recent years have demonstrated the significant correlation between AR and myocardial infarction, as well as cerebrovascular diseases and deaths caused by vascular events, especially the incidence of AR was up to 13.0%-34.0% according to the populations investigated and diverse screening methods [5–9]. AR leads to the failure of effective control of cardiovascular and cerebrovascular diseases, and thus results in repeated attacks and increased risk of fatality. Therefore, increasing attention has been given to this phenomenon in clinical practice.

Mechanisms of AR are likely to be pharmacokinetic or medication adherence issues predominating in majority of aspirin-treated individuals. However, legion potential mechanisms may underlie the phenomenon of AR, such as poor adherence, high platelet turnover due to underlying pathological condition, multiple pathways of platelet activation, drug-drug interaction (e.g. non-steroidal anti-inflammatory drugs and proton pump inhibitors), gene polymorphism, especially in cyclo-oxygenase (COX) 1 and COX-2 [10–11].

AR can be diagnosed in the laboratory by detection of TX function through the production of platelet TXA_2_, such as urinary 11-dehydro-TXB_2_ and serum or plasma TXB_2_, which is largely dependent on platelet COX-1 [12]. TX-dependent platelet function can be also measured by a variety of testing methods, like light or optical aggregation, impedance aggregation, platelet function analyzer-100, rapid platelet function assay, thromboelastography (TEG) and flow cytometry, all of which are associated with clinically important adverse thrombosis events [13]. While the diagnostic criteria are poorly defined for their distinct kinds of revulsants and different dosages. Light transmission aggregometry is considered to be the historical “golden standard” as the validation of AR.

In recent years, Chinese herbal medicine (CHM) is becoming an ideal treatment for AR in China. Researches have found the multitarget intervention effects of CHM for AR. Clinical researches demonstrate that a variety of Chinese herbal compounds promote blood circulation (e.g. composite salvia dropping pill, Tongxinluo capsule, Xuefu Zhuyu decoction, etc.) and certain Chinese herbal extracts or traditional medicine monomers (e.g. ginkgo biloba extract, lumbrokinase, Xinnao Shutong capsule, etc.) can effectively inhibit platelet activity. Laboratorial study demonstrates that salvianolatic acid B is able to inhibit human platelet activation induced by various agonists in vitro by inhibiting phosphodiesterase (PDE) and antagonizing P2Y12 receptor [14]. Cryptotanshinone inhibits the rat platelet aggregation in a concentration dependent manner and also is endowed of Gi-coupled P2Y12 receptor antagonist as demonstrated by docking studies [15]. Xiao-Chai-Hu-Tang inhibits FeCl_3_ induced thrombus formation through inhibition of platelet aggregation, serotonin and TXB_2_ production [16]. Sheshang capsule can be used to treat the coagulopathy induced by Trimeresurus stejnegeri venom through regulating cyclic adenosine monophosphate (cAMP)/protein kinase A (PKA) pathway [17]. Dencichine exerts hemostatic function via aminomethyl phosphonic acid (AMPA) receptors on platelets, thus, facilitating coagulation cascade in a paracrine fashion by control of platelet cytosolic calcium influx, cAMP production and TXA_2_ release [18].

Aspirin is widely used as the primary and secondary prevention of cardiovascular and cerebrovascular disease. Nevertheless, responses to aspirin vary from one patient to another. Recent studies have shown significantly potential of CHM in the antiplatelet therapy. More and more clinical studies had been designed to evaluate the effects of CHM on platelets in patients with higher platelet response correlation disease and to explore the underlying mechanism.

A systematic review without meta-analysis on effectiveness and safety of CHM for AR has been conducted and reported [19]. However, there are still several problems:(1) Different test measures for laboratory AR could lead to different results and also could result in clinical heterogeneity, which was not mentioned in the former systematic review. (2) Some studies with quality problems should be eliminated, because the faulty data would lead to unreliable results. And the systematic review based on the studies with faulty data is meaningless, which could not guide clinical practice. (3) Unreasonable intervention of control group setting: the former systematic review included the trials that used the same CHM in both intervention and control group, which could not objectively reflect the antiplatelet effects of CHM itself in AR. (4) The treatment with syndrome differentiation is one of the characteristics of traditional Chinese medicine (TCM). Different syndromes and treatments cause the clinical heterogeneity of TCM clinical trials. But the former systematic review did not take the influence of syndrome differentiation of TCM in each trial into account. And the former systematic review did not conduct meta-analysis of the effective data either.

Due to all these shortcomings, the former systematic review is insufficient to evaluate the efficiency of TCM for AR. And it has been more than two years since the former systematic review enrolled sixteen randomized controlled trials (RCTs) (before Dec 2012) published. After that, new RCTs with higher methodological qualities investigating TCM in AR treatment have been published.

To apply a very different approach than the previous study, we paid attention to the varied test measures of AR, took the influence of TCM syndrome differentiation into consideration, and tried to do a systematic review and meta-analysis on the base of a very rigorous process of trial selection. In hope that it could accurately evaluate the efficacy and safety of CHM as complementary and alternative medicine in the treatment of AR, and provide evidence for new antiplatelet therapeutics.

## Methods

We reported the results of this systematic review and meta-analysis according to *the Preferred Reporting Items for Systematic Reviews and Meta-Analyses*: *The PRISMA Statement*(S1 Checklist).

### Registration Number

We have registered the protocol of this systematic review and meta-analysis in PROSPERO (available from http://www.crd.york.ac.uk/prospero/display_record.asp?ID=CRD42015020182, S1 Text).

### Eligibility criteria

#### Types of Studies

We only included RCTs investigating the efficacy of CHM for AR, which were published in English or Chinese, regardless of blinding or publication type. But quasi-RCTs studies, which allocated participants according to the date of birth, hospital record number, day of the week and ID number, were excluded. And studies, which did not report any specific outcome data and could not get the intact data from the authors, were excluded.

#### Types of Participants

The participants with AR of any age or gender were included. The most widely used diagnosis test measure of AR was light or optical transmission. Definite items were as following: (1) the platelet aggregation rate (PAR) induced by adenosine diphosphate (ADP) >70%; (2) PAR induced by arachidonic acid (AA) >20%. When meeting one or both of conditions above, it could be diagnosed as AR. Since there was no golden standard diagnosis criterion for AR, we also included other test measures for AR, such as PAR induced by collagen (COL), electrical impedance, serum thromboxane B_2_ (TXB_2_), and semi-automated platelet aggregometry. The exclusion criteria of AR: (1) aspirin allergy; (2) hematological diseases, such as hemorrhagic disorders or hemorrhagic tendency; (3) abnormal platelet count: >450*10^9 or <100*10^9; (4) using effecting platelet aggregation drugs during the last month of the observation period; (5) active gastric ulcer and gastrointestinal bleeding patients; (6) severe liver function damaged patients.

#### Types of Interventions

Studies reported using CHM as monotherapy or adjunctive therapy for AR. CHM was defined as traditional Chinese herb formula, Chinese patent medicine and herbal products extracted from Chinese herbs (e.g. oral liquid, capsule, tablet, pill, powder and injection). There was no limitation on the number of herb use, dosage, frequency, administration, or duration of the treatment. The interventions of control groups: aspirin (regardless of dosage), placebo, other conventional anti-platelet drugs (e.g. dipyridamole, clopidogrel, and cilostazol) used alone or combined with aspirin, or no intervention. But the same CHM used in both of intervention group and control group was excluded.

#### Types of Outcome Measures

The primary outcomes were evaluated by the variation of the PAR induced by AA and/or ADP, and TXB2. The outcomes were measured at the beginning and in the end of the treatment course. The secondary outcomes measurements were clinical efficacy rate and adverse events related to CHM. The standard of clinical efficacy: (1) AR becomes aspirin semi-resistance (ASR) or aspirin sensitive; (2) ASR becomes aspirin sensitive. The diagnosis criteria of aspirin sensitive: ≤70% PAR induced by ADP and ≤20% PAR induced by AA.

### Literature Search

Comprehensive search terms for relevant studies were performed in the following electronic databases: English databases included the Cochrane Central Register of Controlled Trials (CENTRAL, OVID), PubMed (OVID), and EMBASE (OVID), Chinese databases included China National Knowledge Infrastructure (CNKI), Chinese Biomedicine Database (CBM, SinoMed), Chinese Science and Technology Periodical Database (VIP) and WanFang Database. The search duration was inception of the databases to March 2015. Search terms combined “aspirin resistance or aspirin non-responders” and “traditional Chinese medicine or Chinese herbal medicine or Chinese medicine or Chinese herbal drug or traditional herbal medicine or traditional East Asian medicine or integration of Chinese herbal and Western medicine” and “randomized controlled trials”.

### Study Selection and Data Extraction

Two investigators (Chen HY and Shen ZJ) independently selected the eligible trials. Data were also independently extracted from the included trials using a pre-designed standard data extract form by two investigators, including demographic and clinical characteristics of participants, methods, interventions and outcomes. If the outcome data of relevant studies were unclear or missing, we sought further information from the authors. Different opinions between the two investigators were settled through discussion or consultation with the third party (Chen JD).

### Risk of Bias in Included Studies

Two investigators independently assessed the risk of each individual study by using the risk of bias assessment tool from Cochrane Handbook for Systematic Review (Version 5.1.0). Disagreements resolved by discussion or consultation with the third party. The following items were assessed for each study: random sequence generation, allocation concealment, blinding of participants and personnel, blinding of outcome assessments, incomplete outcome data, selective reporting, and other sources of bias. Quality of each item was divided into low/unclear/high risk of bias. We also planned to perform a funnel plot to test publication bias, if more than 10 trials were included.

### Data Analysis

We used Review Manager 5.3 software to analyze data. Heterogeneity was assessed by using both the Chi^2^ test and the I^2^ test. If no statistical heterogeneity existed in pooled studies (P>0.1, I^2^≤50%), we adopted a fixed-effect model for meta-analysis, otherwise a random-effect model was applied (P<0.1, I^2^>50%). In consideration of the differences in participants (e.g. demographic characteristics, primary disease), interventions (e.g. mode, dose, duration), outcomes (e.g. determination method, diagnosis criteria), the subgroup analysis was planned to conduct.

## Results

### Study Selection

**Fig 1:**
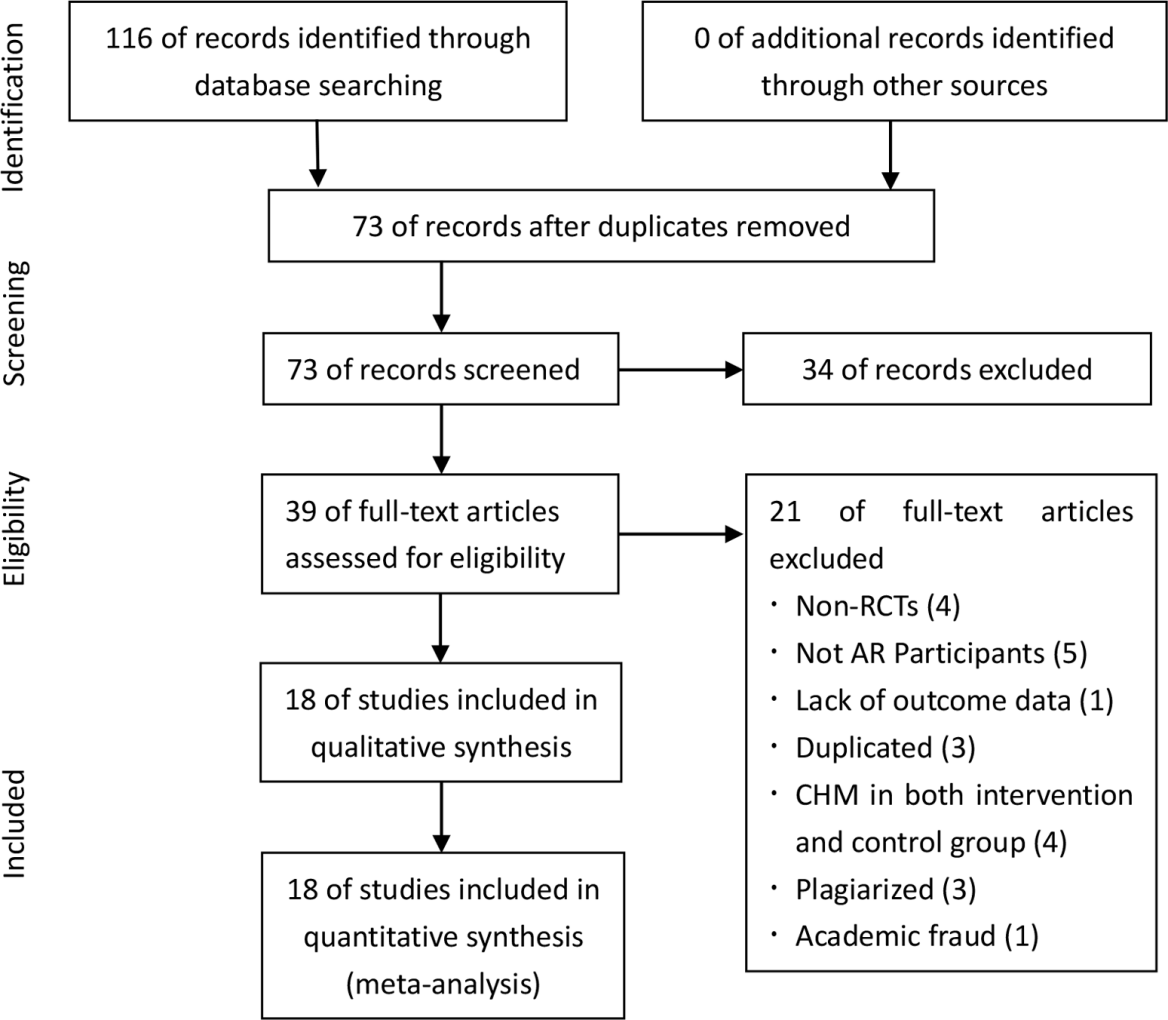
PRISMA Flow Chart.

A total of 116 studies were identified through searching 7 electronic databases. After duplication removed, 73 records remained. After screening titles and abstracts, 34 records were excluded. Among the 34 excluded studies, 7 studies were non-RCTs, 9 papers were reviews, 5 using the same CHM in both intervention and control group, 2 were experiment researches, and 11 were not participants with AR. By reading the full text, 18 studies remained. Among those removed 21 studies, 4 were excluded for non-RCTs, 5 were not AR participants, 1 was lack of specific outcome data, 3 were duplicated, 4 used the same CHM in both intervention and control group, 3 were plagiarized, and 1 was removed due to academic fraud. Finally, 18 eligible studies were included for this systematic review and meta-analysis. A PRISMA flow chart depicted the search process and study selection (Fig 1).

### Study Characteristics

A sum of 1,460 patients was enrolled in 18 studies [20–37] (8 patients withdraw or dropped out), at which 622 patients participated in CHM combined with aspirin, 110 patients used CHM as monotherapy, and 728 patients received aspirin alone therapy or the addition of other anti-platelet drugs to aspirin therapy. The sample size ranged from 30 to 180, the average was 81.11 per trial. The mean age of participants ranged from 12.31 to 68.5 years old, the majority of trials reporting a mean age except 5 trials only reporting the age range. And all trials had a minimum of 2 weeks’ treatment duration, while some of them were up to 12 weeks.

Of the 18 included trials in this systematic review, only 1 trial (Wang J 2012) was performed randomized, single center, double-blinded, and 7 trials (Song SJ 2008, Chai ZQ 2008, Yin CH 2010, Zhang JL 2010, Liu YJ 2013, Chen G 2013, Liu DF 2015) were RCTs with 3 arms. All trials were carried out in China and published in Chinese. The primary disease of these trials included coronary heart disease (n = 6), cerebral infarction (n = 8), hypertension (n = 1), and other cardiovascular disease (n = 3).

12 trials used light or optical transmission to test the PAR, 1 trial (Yin CH 2010) applied electrical impedance to measure the rate, one (Wang J 2012) used thrombelastogram, and 4 trials did not report their test measures. The major trials adopted an average PAR ≥70% with 10μmol/L ADP and/or ≥20% induced by 0.5mmol/L AA as AR diagnosis criteria. The AR diagnosis criterion of 4 trials (Wang J 2012, Su WJ 2012, Zhang L 2013, Tu XP 2013) was an average PAR ≥70% with 10μmol/L ADP and/or ≥20% induced by 0.5mg/mL AA. And 1 trial (Yin CH 2010) adopted a platelet aggregation rate induced by 10μg/mL ADP ≥13ohm or 2μg/mL COL ≥18ohm as the diagnosis criteria of AR.

In 18 trials, the intervention group of 16 trials used CHM combined with regular aspirin (100mg/d) treatment, 5 trials (4 were 3 arms trials) used CHM as monotherapy for intervention group, and 1 trail (Wang J 2012) used CHM combined with clopidogrel treatment. 10 trials adopted the regular aspirin therapy (100mg/d) as the control group, 2 trials severally adopted the higher dose aspirin therapy (200mg/d, 300mg/d) as the control group, and 6 trials’ control group was the aspirin combined with other anti-platelet drugs (dipyridamole 150mg/d n = 4, clopidogrel 75mg/d n = 2). CHM adjunct therapy was compared with regular aspirin treatment in 10 trials and compared with the addition of other anti-platelet drugs in 6 trials. And in 5 CHM monotherapy trials, 2 trials (Su WJ 2012, Chen G 2013) adopted CHM monotherapy compared with higher dose aspirin treatment, and the rest 3 trials compared with the regular aspirin treatment.

All trials used PAR as the primary outcome, with all trials reported the PAR induced by ADP, and 14 trials reported the PAR induced by AA. Among them, TXB_2_ was reported at the same time in 5 trials. However, only 2 trials (Liu QK 2010, Su WJ 2012) observed the clinical effective rate. Adverse events were reported in 7 trials, bleeding events were reported in 5 trials, and cerebral infarction was observed in 4 trials. The detailed characteristics of included trials and the contents of CHM in the included trials were listed in Tables 1 and 2.

**Table 1:** Characteristics of Included Studies.

Control Group Method	Included Trials	Primary Disease	Diagnosis Criteria of AR	Test Measures of AR	Sample (male/ female)		Age (range/mean)	Interventions		Outcomes(* P<0.05, #P<0.01)
					Intervention	Control	(year)	Intervention	Control	
**C:Aspirin100mg/d**	**Song SJ 2008**^**[**^^**20**^^**]**^	Coronary heart disease	AA# and ADP#	Unclear	I1:24 (7/17);	23(6/17)	I1:65.46±8.08;	I1:Tongxinluo capsule,3pieces,tid+aspirin,100mg/d,4 weeks;	Aspirin,100mg/d,4 weeks	①②③*⑧*
					I2:4(7/16)		I2:6.17±7.94;	I2:clopidogrel,75mg/d+aspirin,100mg/d,4 weeks		
							C:65.23±7.25			
	**Zhang JL 2010**^**[**^^**24**^^**]**^	Cerebral infarction	ADP†	Light or optical transmission	I1:21;	20		I1:Tongxinluo capsule,4pieces,tid+aspirin,100mg/d,1 month;	Aspirin,100mg/d,1 month	①* ③*
					I2:19			I2:cilostazol,100md/d+aspirin,100mg/d,1 month		
	**Yin CH 2010**^**[**^^**23**^^**]**^	Coronary heart disease	ADP§ and COL	Electrical impedance	I1:30(18/12);	29(17/12)	I1:43-82/66.55±10.82;	I1:Tongxinluo capsule,3pieces,tid+aspirin,100mg/d,1 month;	Aspirin,100mg/d,1 month	①* ④*⑮⑰
**C:Aspirin 100mg/d**					I2:30(19/11)		I2:46-84/66.69±10.56;	I2:Tongxinluo capsule,3 pieces,tid,1 month		
							C:44-82/66.93±10.75			
	**Chai ZQ 2008**^**[**^^**21**^^**]**^		AA# and ADP#	Light or optical transmission	I1:10(3/7);	10(3/7)	I1:67.40±14.70;	I1:Compound danshen dripping pill,270mg,tid+aspirin,100mg/d,2 weeks;	Asprin,100mg/d,2 weeks	①*②*
					I2:10(4/6)		I2:12.31±17.61;	I2:Compound danshen dripping pill,270mg,tid,2 weeks		
							C:67.70±12.79			
**C:Aspirin 100mg/d**	**Guo HY 2012**^**[**^^**30**^^**]**^		ADP#	Light or optical transmission	50	53		Compound danshen dripping pill,270mg,tid+aspirin,100mg/d,1 month	Aspirin,100mg/d,1 month	①#⑬*⑭*⑰
	**Zhang X 2013**^**[**^^**33**^^**]**^	Coronary heart disease	AA# and ADP#	Light or optical transmission	20(10/10)	19(10/9)	I:55.7±6.8;	Compound danshen dripping pill,270mg,tid+aspirin,100mg/d,1 month	Aspirin,100mg/d,1 month	①#②⑨⑮⑯
							C:57.7±8.2			
	**Wu TH 2012**^**[**^^**28**^^**]**^		AA# and ADP#	Light or optical transmission	30	30	35-80(total)	Xufuzuyu Decoction,1dose/d+aspirin,100mg/d,4 weeks	Aspirin,100mg/d,4 weeks	①*②*
	**Liu YJ 2013**^**[**^^**31**^^**]**^	Cerebral infarction	AA# and ADP#	Light or optical transmission	I1:50;	42	75.19±10.47(total)	I1:Sanqi Tongshu Capsule,200mg,tid+aspirin,100mg/d,1 month;	Aspirin,100mg/d,1 month	①*②*③*⑩*
**C:Aspirin 100mg/d**					I2:48			I2:cilostazol,100md/d+aspirin,100mg/d,1 month		
	**Huang HM 2014**^**[**^^**36**^^**]**^	Cerebral infarction	AA# and ADP#	Unclear	49(29/20)	45(27/18)	I:68.7±6.5;	Naoshuantong capsule,1.2,tid+aspirin,100mg/d,12 weeks	Aspirin,100mg/d,12 weeks	①*②*⑪*⑫*⑬*
							C:68.5±6.3			
	**Liu DF 2015**^**[**^^**37**^^**]**^	Coronary heart disease	AA# and ADP#	Light or optical transmission	I1:20(11/9);	20(9/11)	44-78(total)	I1:Huxin capsule,2 pieces,tid,21 days;	Aspirin,100mg/d,21 days	①*②*⑮
					I2:20(8/12)			I2:Huxin capsule,2 pieces,tid+aspirin,100mg/d,21 days		
**C:Aspirin 200mg/d**	**Chen G 2013**^**[**^^**32**^^**]**^	Coronary heart disease	AA# and ADP#	Light or optical transmission	I1:20(11/9);	18(10/8)	I1:64.9±8.6;	I1:Xueshuanxinmaining capsule,4 pieces,tid+aspirin,100mg/d,4 weeks;	Aspirin,200mg/d,4 weeks	①*②*⑮
					I2:20(11/9)		I2:6.4.9±8.6;	I2:Xueshuanxinmaining capsule,4 pieces,tid,4 weeks		
							C:65.7±7.8			
**C:Aspirin 300mg/d**	**Su WJ 2012**^**[**^^**27**^^**]**^		AA§ and ADP$	Light or optical transmission	30(15/15)	30(13/17)	I:43-70/62;	Diaoxinxuekang capsule,1.6g,tid,4 weeks	Aspirin,300mg/d,4 weeks	①*②*③⑤* ⑦*⑮# ⑰
							C:41-70/61.2			
**Dipyridamole 150mg/d+aspirin 100mg/d**	**Liu HQ 2008**^**[**^^**22**^^**]**^	Cerebral infarction	ADP†	Unclear	40(21/19)	40(23/17)	I:38–72;	Zuyu Tongmai Capsule,2pieces,tid+aspirin,100mg/d,1 month	Dipyridamole,150mg/d+aspirin,100mg/d,1 month	①#
							C:41–75			
	**Liu QK 2010**^**[**^^**25**^^**]**^	Cerebral infarction	AA# and ADP#	Light or optical transmission	36(21/15)	36(18/18)	I:46-78/65;	I1:Gingko biloba tablet,2pieces,tid+aspirin,100mg/d,1 month	Dipyridamole,150mg/d+aspirin,100mg/d,1 month	①#②#⑦*⑮# ⑰
							C:49-78/67			
	**Ma JX 2012**^**[**^^**29**^^**]**^	Cerebral infarction	AA# and ADP#	Unclear	40(23/17)	40(21/19)	I:43-76/63;	Sodium ferulate tablets,100mg,tid+aspirin,100mg/d,4 weeks	Dipyridamole,150mg/d+aspirin,100mg/d,4 weeks	①*②*③*
							C:45-78/65			
	**Tu XP 2013**^**[**^^**35**^^**]**^	Cerebral infarction	AA§ and ADP†	Light or optical transmission	50(29/21)	50(26/24)	I:48-71/61.98±7.92;	Compound danshen dripping pill,270mg,tid+aspirin,100mg/d,1 month	Dipyridamole,150mg/d+aspirin,100mg/d,1 month	①*②*⑬* ⑮*
							C:49-70/61.55±8.12			
**Clopidogrel, 75mg/d+aspirin,100mg/d**	**Wang J 2012**^**[**^^**26**^^**]**^	Coronary heart disease	AA† and ADP#	Thrombelast-ogram	90(50/40)	90(61/29)	I:62.4±13.1;	Puerarin injection,200mg/d+clopidogrel, 75mg/d,7 days	Clopidogrel, 75mg/d+aspirin,100mg/d,7 days	①②#⑥*
							C:65.1±17.0			
	**Zhang L 2013**^**[**^^**34**^^**]**^	Cerebral infarction	AA§ and ADP¶	Light or optical transmission	42(24/18)	42(21/21)	I:44-86/52±13;	Buyang Huanwu Decoction,1 dose/d+aspirin,100mg/d,90 days	Clopidogrel, 75mg/d+aspirin,100mg/d,90 days	①②⑬⑰
							C:48-82/53±11			

Diagnosis Criteria of AR: AA#: 0.5mmol/L arachidonic acid induced PAR≥20%; ADP#: 10μmol/L adenosine diphosphate induced PAR≥70%; ADP†: 10mmol/L adenosine diphosphate induced PAR≥70%; ADP§: 10μg/mL adenosine diphosphate induced PAR≥13ohm; COL: 2μg/mL collagen induced PAR≥18ohm; AA†: 5*10^-3mg/mL arachidonic acid induced PAR>50%; AA§: 0.5g/L arachidonic acid induced PAR≥20%; ADP$: 1.0mmol/L adenosine diphosphate induced PAR≥70%; ADP¶: 110mmol/L adenosine diphosphate induced PAR≥70%.

Outcomes: ①ADP*18 ②AA*14 ③TXB_2_*5 ④COL*1 ⑤6-K-PGF1α*1 ⑥11-dTXB_2_*1 ⑦Clinical effective rate*2 ⑧CRP*1 ⑨hs-CRP*1 ⑩IL-6*1 ⑪NHISS score*1 ⑫BI score*1 ⑬Cerebral infarction reoccurrence rate*4 ⑭Acute myocardial infarction rate*1 ⑮Adverse events*7 ⑯hepar and renal function*1 ⑰Bleeding Events*5

ADP: PAR induced by adenosine diphosphate; AA: PAR induced by arachidonic acid; TXB_2_: thromboxane B_2_; COL: PAR induced by collagen; 6-K-PGF1α: 6-keto-prostaglandin F1α; 11-dTXB_2_: urinary 11-dehydro thromboxane B_2_; CRP: C response protein; hs-CRP: high-sensitivity C-reactive protein; IL-6: interleukin-6; NHISS: National Institutes of Health Stroke Scale; BI: Barthel Index.

**Table 2:** Content of CHM.

Included studies	CHM herbal medicine	Content(Chinese pinyin name,Latin herb name)	Preparation style	Dosage	Chinese patent medicine
**Song SJ 2008;Yin CH 2008;Zhang JL 2010**	Tongxinluo capsule	Renshen(*Panax ginseng* C. A. Meyer),Shuizhi(*Whitmania pigra* Whitman or *Hirudo nipponica* Whitman or *Whitmannia acranulata* Whitman),Quanxie(*Bulhus martensii* Karsch),Chishao *Paeonia lactiflora* Pall. or *Paeonia veitchii* Lynch),Chantui(*Cryptotympana pustulata* Fabricius),Tubiecong(*Eupolyphaga Sinesis* Walker or *Steleophaga plancyi*(Boleny),Wugong(*Scolopendra subspinipes mutilans* L. Koch),Tanxiang(*Santalum album* L.),Jiangxiang(*Dalbergia odorifera* T.Chen),Ruxiang(*Boswellia carterii* Birdw. or *Boswellia bhaw-dajiana* Birdw.),Suanzaoren(*Ziziphus jujuba* Mill. var. *spinosa* (Bunge) Hu ex H.F.Chou),Bingpian(*Cinnamomum camphora* (L.) Presl)	Capsule	2–4 capsules, tid	Yes
**Chai ZQ 2008;Guo HY 2012;Zhang X 2013;Tu XP 2013**	Compound danshen dripping pill	Danshen(*Salvia miltiorrhiza* Bge.),Sanqi(*Panax notoginseng* (Burk.) F. H. Chen),Bingpian(*Cinnamomum camphora* (L.) Presl)	Dripping pill	10 pills, tid	Yes
**Liu HQ 2008**	Zhuyu Tongmai capsule	Shuizhi(*Whitmania pigra* Whitman or *Hirudo nipponica* whitman or Whitmania acranulata Whitman),Mengcong(Tabanus mandarinus Schiner or Atylotus bivittateinus Takahasi),Taoren(*Prunus persica* (L.) Batsch or *Prusua davidiana* (Carr.) Franch.),Dahuang(*Rheum palmatum* L. or *Rheum tanguticum* Maxim.ex Balf. or *Rheum offcihale* Baill.)	Capsule	2 capsules, tid	Yes
**Liu QK 2010**	Gingko biloba tablet	Extract of Yingxingye(*Ginkgo biloba* L.)	Tablet	2 tablets, tid	Yes
**Wang J 2012**	Puerarin injection	Extract of Gegeng(*Pueraria lobata* (Willd.)Ohwi)	Injection	200–400mg,qd	Yes
**Su WJ 2012**	Diaoxinxuekang capsule	Extract of Huangshanyao(*Dioscorea panthaica* Prain et Burk. or *Dioscorea nipponica* Makino)	Capsule	1–2 capsules, tid	Yes
**Wu TH 2012**	Xufuzuyu Decoction	Danggui(*Angelica sinensis* (Oliv.)Diels.)9g,Dihuang(*Rehmannia glutinosa* Libosch.)9g,Taoren(*Prunus persica* (L.) Batsch or Prusua davidiana (Carr.) Franch.)12g,Honghua(Carthamus tinctorius L.)9g,Zhiqiao(Citrus aurantium L.)6g,Chishao(*Paeonia lactiflora* Pall. or *Paeonia veitchii* Lynch)6g,Chuanxiong(*Ligusticum chuanxiong* Hort.)5g,Chaihu(*Bupleurum chinense* DC. or *Bupleurum scorzonerifolium* Willd.)3g,Jiegeng(*Platycodon grandiflorum* (Jacq.)A.DC.)5g,Niuxi(*Achyranthes bidentata* B1.)9g,Gancao(*Glycyrrhiza uralensis* Fisch. or *Glycyrrhiza inflata* Bat. or *Glycyrrhiza glabra* L.)3g	Decoction	1 dose, qd	No
**Ma JX 2012**	Sodium ferulate tablets	Extract of Chuanxiong(*Ligusticum chuanxiong* Hort.)	Tablet	2–4 capsules, tid	Yes
**Liu YJ 2013**	Sanqi Tongshu Capsule	Extract of Sanqi(Panax notoginseng (Burk.) F. H. Chen)	Capusle	1 capsule, qd	Yes
**Chen G 2013**	Xueshuanxinmaining capsule	Chuanxiong(*Ligusticum chuanxiong* Hort.),danshen(Danshen(*Salvia miltiorrhiza* Bge.),Shuizhi(*Whitmania pigra* Whitman or *Hirudo nipponica* Whitman or *Whitmannia acranulata* Whitman), Maodongqing(*Ilex pubescens* Hook. et Arn. var. pubescens),Niuhuang(*Bostaurus domesticus* Gmelin),Shexiang(*Moschus berezovskii* Flerov or *Moschus sifanicus* Przewalski or *Moschus moschiferus* Linnaeus),Huaihua(*Sophora japonica* L.),Renshen Jingye Zongzaogan(*Panax ginseng* C.A.Mey.),Bingpian(*Cinnamomum camphora* (L.) Presl),Chansu(*Bufo bufo gargarizans* Cantor or *Bufo melanostictus* Schneider)	Capsule	4 capsules, tid	Yes
**Zhang L 2013**	Buyang Huanwu Decoction	Huangqi(*Astragalus membranaceus* (Fisch.)Bge.var.*mongholicus* (Bge.) Hsiao or *Astragalus membranaceus*(Fisch.)Bge.)15g,Dangguiwei(*Angelica sinensis* (Oliv.)Diels.)12g,Chishao(*Paeonia lactiflora* Pall. or *Paeonia veitchii* Lynch)12g,Dilong(*Pheretima aspergillum*(E.Perrier) or *Pheretima vulgaris* Chen or *Pheretima guillelmi*(Michaelsen) or *Pheretima Pectinifera* Michaelsen)10g,Chuanxiong(*Ligusticum chuanxiong* Hort.)9g,Taoren(*Prunus persica* (L.) Batsch or *Prusua davidiana* (Carr.) Franch.)10g,Honghua(*Carthamus tinctorius* L.)10g,Zhishi(*Citrus aurantium* L. or *Citrus sinensis* Osbeck)6g	Decoction	1 dose, qd	No
**Huang HM 2014**	Naoshuantong capsule	Puhuang(*Typha angustifolia* L.),Chishao(*Paeonia lactiflora* Pall.or *Paeonia veitchii* Lynch),Yujin(*Curcuma wenyujin* Y.H.Chen et C.Ling or *Curcuma longa* L.or *Curcuma kwangsiensis* S.G.Lee et C.F.Liang or *Curcuma phaeocaulis* Val.),Tianma(*Gastrodiaelata* B1.),Loulu(*Rhaponticum uniflorum* (L.) DC.)	Capsule	3 capsules, tid	Yes
**Liu DF 2015**	Huxin capsule	Huangqi(*Astragalus membranaceus* (Fisch.)Bge.var.*mongholicus* (Bge.) Hsiao or *Astragalus membranaceus*(Fisch.)Bge.),Hupo(*Ambrum*),Guizhi(*Cinnamomum cassia* Presl),Gancao(*Glycyrrhiza uralensis* Fisch.or *Glycyrrhiza inflata* Bat.or *Glycyrrhiza glabra* L.),Longgu(OsDraconis(*FossiliaOssiaMastodi*),Muli(*Ostrea gigas* Thunberg or *Ostrea talienwhanensis* Crosse or *Ostrea rivularis* Gould),Sanqi(*Panax notoginseng* (Burk.) F. H. Chen),Shuizhi(*Whitmania pigra* Whitman or *Hirudo nipponica* Whitman or *Whitmannia acranulata* Whitman),Honghua(*Carthamus tinctorius* L.),Danshen(*Salvia miltiorrhiza* Bge.),Chishao(*Paeonia lactiflora* Pall. or *Paeonia veitchii* Lynch)	Capsule	2 capsules, tid	Yes

qd: once a day; bid: twice a day; tid: three times a day.

### Assessment of risk of Bias

**Fig 2:**
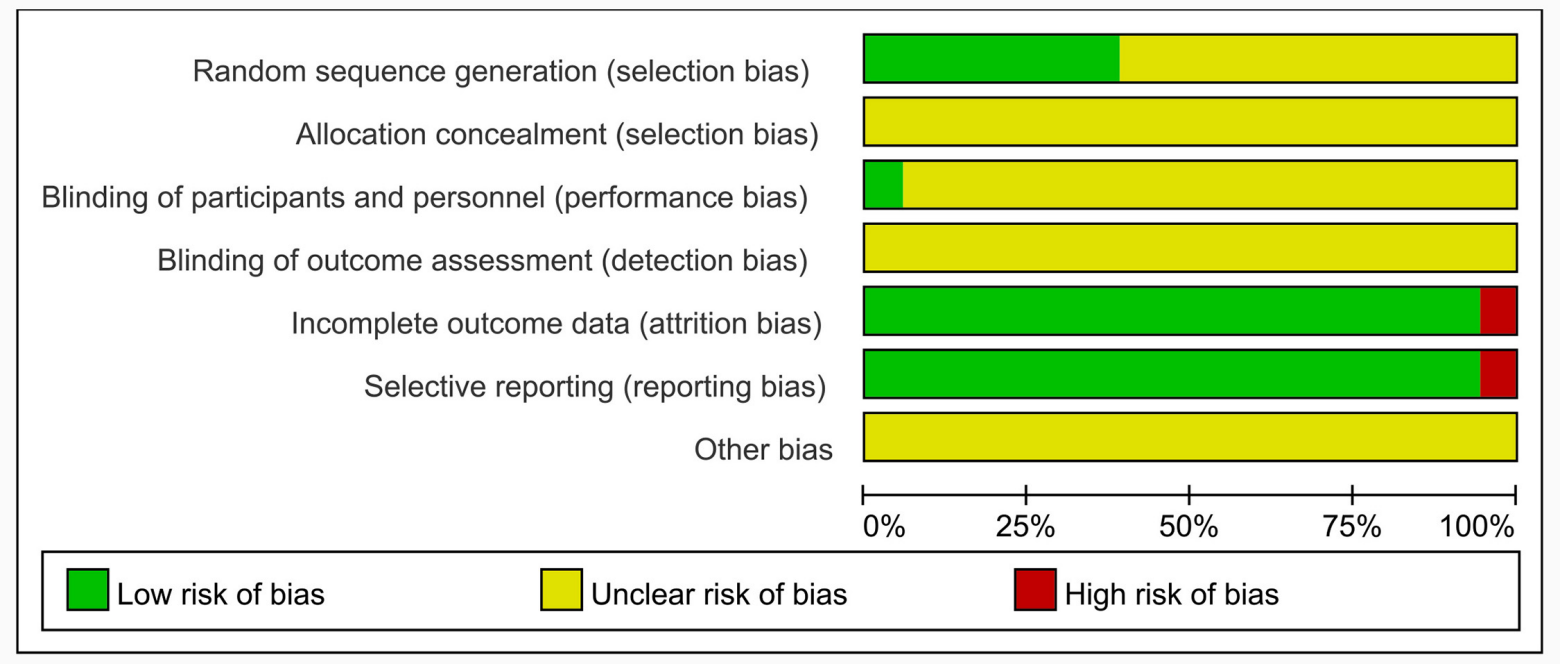
Risk of Bias Graph.

**Fig 3:**
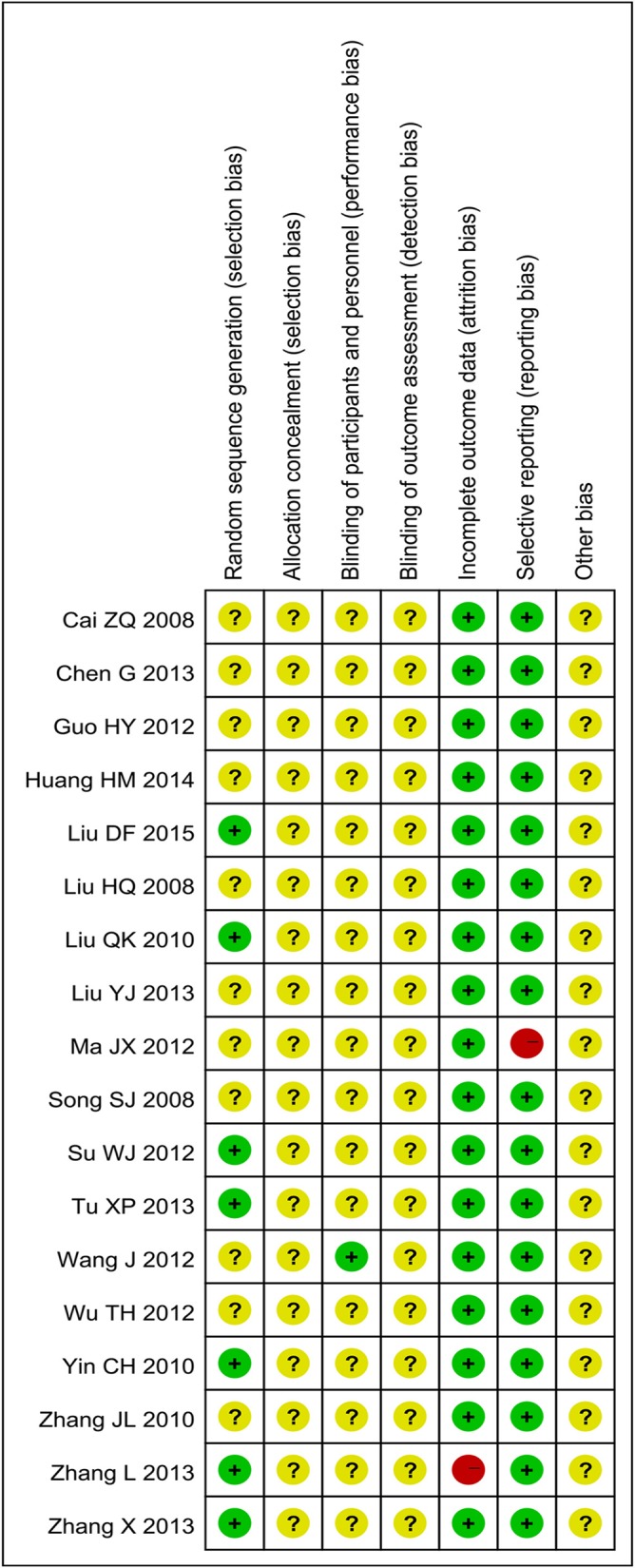
Risk of Bias Summary.

We tried to get further information about some included trials from authors by E-mail, unfortunately there was no response. The methodological quality of the whole trials was generally poor (Figs 2 and 3). Although all trials claimed that they were RCTs, only 7 articles reported the appropriate method of random sequence generation, and all of them adopted the random number table method. The allocation concealment was not mentioned in all trials. Only 1 trail (Wang J 2012) reported the blinding of participants and personnel, but none of included trials mentioned blinding of outcome assessment. 8 patients were reported withdraw or dropped out in 1 trail (Zhang L 2013) due to some reasons. Intention-to-treat (ITT) analysis was not conducted in that trail. Pre-designed outcomes were all reported except 1 trail (Ma JX 2012). Moreover, the sample size of all trials was small and none of included trials provided a pretrial estimation of sample size. In a word, the methodological quality of most included trials was poor.

### Results of Individual Studies

#### 1 PAR induced by ADP

**Fig 4:**
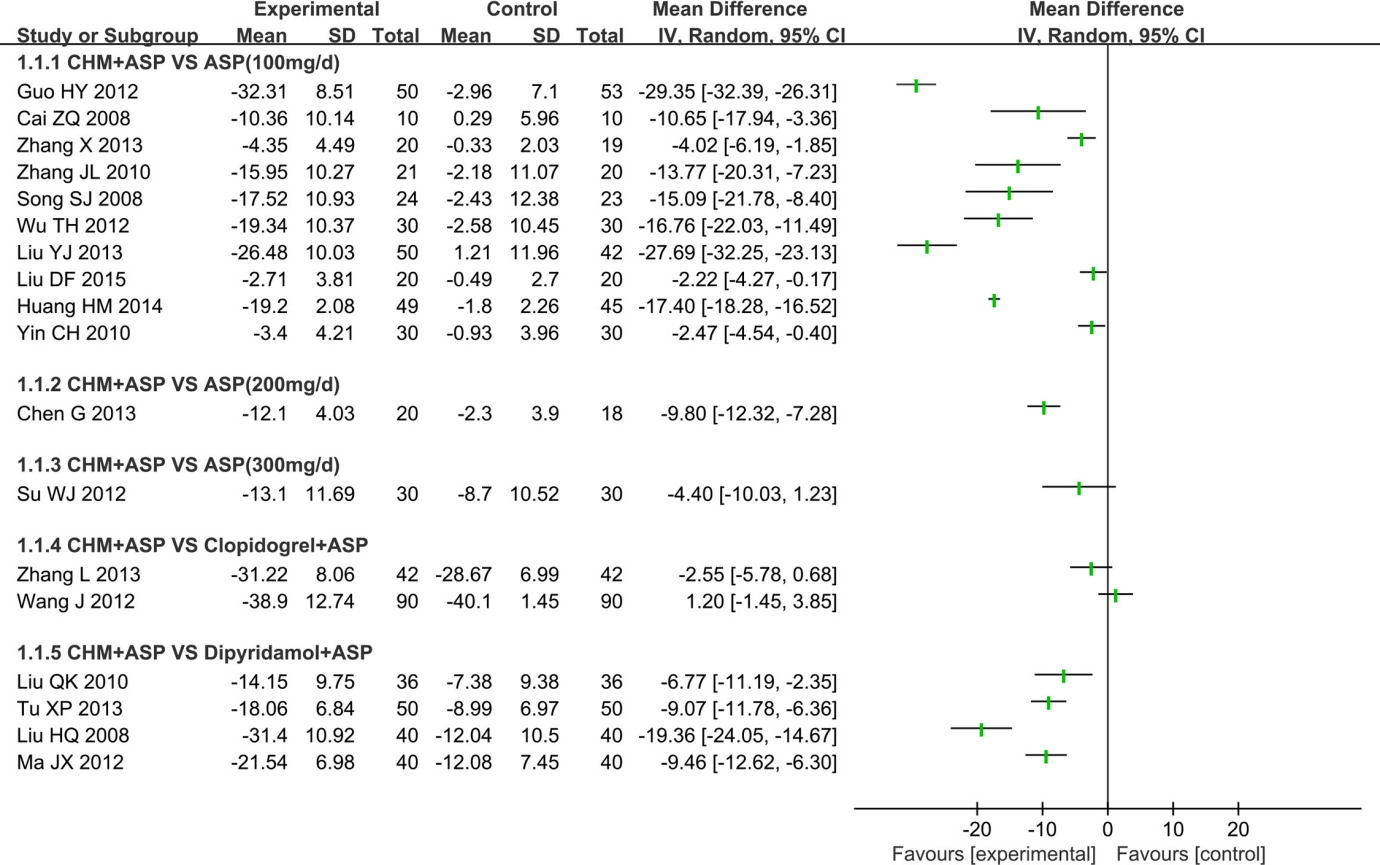
Platelet Aggregation Rate Induced by ADP.

All included trials reported the PAR induced by ADP, 10 of them adopted CHM plus aspirin versus regular dose aspirin (100mg/d), 2 of them severally adopted CHM plus aspirin versus high dose aspirin (200mg/d, 300mg/d), 2 of them adopted CHM plus aspirin versus clopidogrel plus aspirin, and 4 of them adopted CHM plus aspirin versus dipyridamole plus aspirin (Fig 4).

**1.1 CHM plus Aspirin versus Regular Aspirin Therapy:** 10 RCTs compared CHM plus Aspirin with regular aspirin treatment (100mg/d), 7 of them used light or optical transmission to test the PAR, one (Yin CH 2010) used electrical impedance to test the rate and two didn’t declare test methods. All RCTs reported that there was significant difference of effect of CHM plus aspirin to reduce the PAR induced by ADP (P<0.05) compared with regular aspirin therapy except Song SJ 2008.

There were 6 types of CHM used in these 10 RCTs (①Tongxinluo Capsule, ②Compound danshen dripping pill, ③Xufuzuyu Decoction, ④Sanqi Tongshu Capsule, ⑤Naoshuantong capsule, ⑥Huxin capsule). Tongxinluo capsule was involved in 3 trails, but 2 trials of them used different test measures to test the PAR and the rest one did not report the type of test measure. Compound danshen dripping pill was also involved in 3 trials and all these 3 trials used light or optical transmission to test the PAR. The other 4 types of CHM were separately reported in 4 trials.

In consideration of clinical heterogeneity in terms of participants, varieties of CHM and the testing measures, we couldn’t perform a meta-analysis. But the PAR induced by ADP tended to be effective (Fig 4). And we tried to perform subgroup analysis of the Compound danshen dripping pill, which adopted the same intervention method and also used the same test measures of PAR.

**Fig 5:**
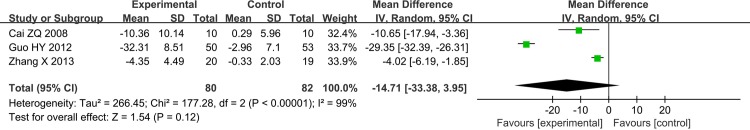
Platelet Aggregation Rate Induced by ADP (Compound Danshen Dripping Pill+ ASP vs ASP, ASP: Aspirin 100mg/d).

**1.1.1 Compound Danshen Dripping Pill plus Aspirin versus Aspirin:** 3 RCTs made a comparison of Compound danshen dripping pill plus aspirin with aspirin (100mg/d) and all of them reported the PAR induced by ADP was significant decreased (P<0.05). However, the meta-analysis showed there was significant heterogeneity in the studies with PAR induced by ADP (Chi^2^ = 177.28, I^2^ = 99%, P<0.00001) (Fig 5).

**1.2 CHM plus Aspirin versus High-dose Aspirin Therapy:** 1 RCT (Chen G 2013) reported there was significant effect of reducing the PAR induced by ADP in comparison of the CHM monotherapy versus aspirin 200mg/d (P<0.05). Another RCT (Su WJ 2012) also claimed that there was significant difference in the reduction of PAR induced by ADP when CHM monotherapy was compared with 300mg/d aspirin therapy (P<0.05).

**1.3 CHM plus Aspirin versus Clopidogrel plus Aspirin:** 2 RCTs compared CHM plus aspirin with clopidogrel plus aspirin and one (Wang J 2012) used the thrombelastogram to test the PAR. Both of them reported significant difference of decrease in the PAR induced by ADP of CHM plus aspirin compared with that before treatment (P<0.05), but they did not report the comparison of control group. Due to the varieties of CHM and test measures, we failed to perform a meta-analysis.

**1.4 CHM plus Aspirin versus Dipyridamole plus Aspirin:** There were 4 RCTs compared CHM plus aspirin with dipyridamole. All these RCTs showed significant difference of decrease in the PAR induced by ADP (P<0.05). All RCTs adopted different CHM plus aspirin and 2 RCTs (Liu HQ 2008 and Ma JX 2012) did not report the test measures, so the meta-analysis could not be performed either.

#### 2 PAR induced by AA

**Fig 6:**
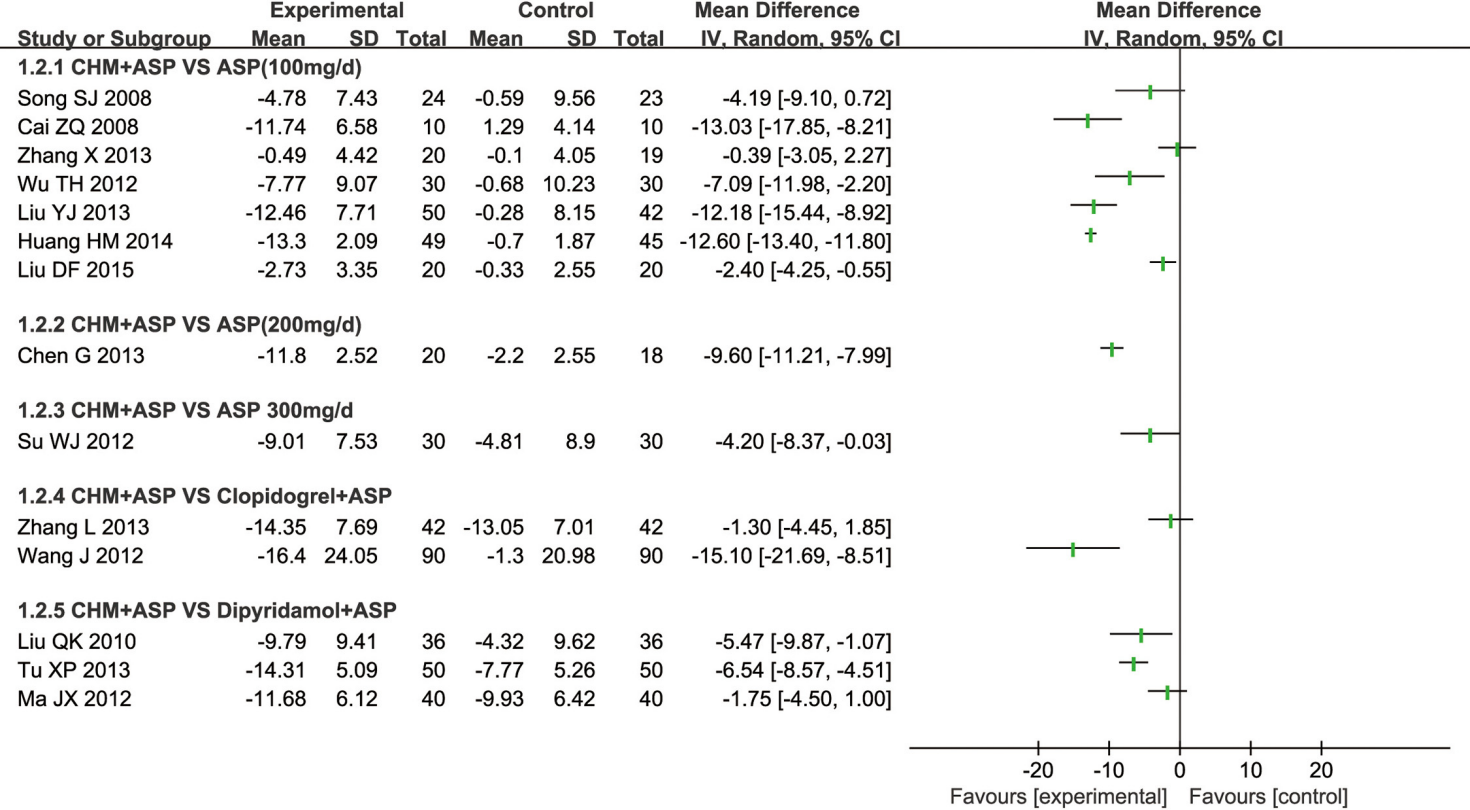
Platelet Aggregation Rate Induced by AA.

14 trials reported the PAR induced by AA, 7 of them compared CHM plus aspirin with regular dose aspirin (100mg/d), 2 of them severally compared CHM plus aspirin with high dose aspirin (200mg/d, 300mg/d), 2 of them compared CHM plus aspirin with clopidogrel plus aspirin, and 3 of them compared CHM plus aspirin with dipyridamole plus aspirin (Fig 6).

**2.1 CHM plus Aspirin versus Regular Aspirin Therapy:** 7 RCTs reported the PAR induced by AA, 5 of them used light or optical transmission to test the PAR and 2 of them (Liu QK 2008 and Ma JX 2012) did not report the test measures. 6 different types of CHM were used in 7 RCTs (①Tongxinluo Capsule, ②Compound danshen dripping pill, ③Xufuzuyu Decoction, ④Sanqi Tongshu Capsule, ⑤Naoshuantong capsule, ⑥Huxin capsule). All RCTs reported significant effect of CHM plus aspirin to reduce the PAR induced by AA (P<0.05) compared with aspirin 100mg/d treatment except Song SJ 2008 and Zhang X 2013. We tried to perform a meta-analysis of Compound danshen dripping pill plus aspirin.

**Fig 7:**
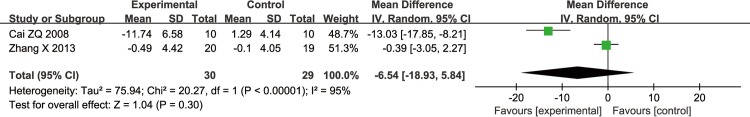
Platelet Aggregation Rate Induced by AA (Compound Danshen Dripping Pill +ASP vs ASP, ASP: Aspirin 100mg/d).

**2.1.1 Compound Danshen Dripping Pill plus Aspirin versus Aspirin:** 2 RCTs reported that the PAR induced by AA in patients with Compound danshen dripping pill plus aspirin was significantly decreased in these RCTs (P<0.05). However, the meta-analysis showed there was significant heterogeneity in the studies with PAR induced by AA (Chi^2^ = 20.27, I^2^ = 95%, P<0.00001) (Fig 7).

**2.2 CHM plus Aspirin versus High-dose Aspirin Therapy:** Both Chen G 2013 and Su WJ 2012 reported there was significant effect of reducing the PAR induced by AA in comparison of the CHM plus aspirin versus aspirin (P<0.05).

**2.3 CHM plus Aspirin versus Clopidogrel plus Aspirin:** 2 RCTs (Wang J 2012 and Zhang L 2013) adopted CHM plus aspirin versus clopidogrel plus aspirin, and one (Wang J 2012) reported there was significant difference of reducing the PAR induced by AA (P<0.05).

**2.4 CHM plus Aspirin versus Dipyridamole plus Aspirin:** 3 RCTs showed significant difference of decrease in the PAR induced by AA (P<0.05). We could not perform a meta-analysis, because 3 RCTs adopted various CHM and one (Ma JX 2012) did not report the test measure.

#### 3 TXB_2_

**Fig 8:**
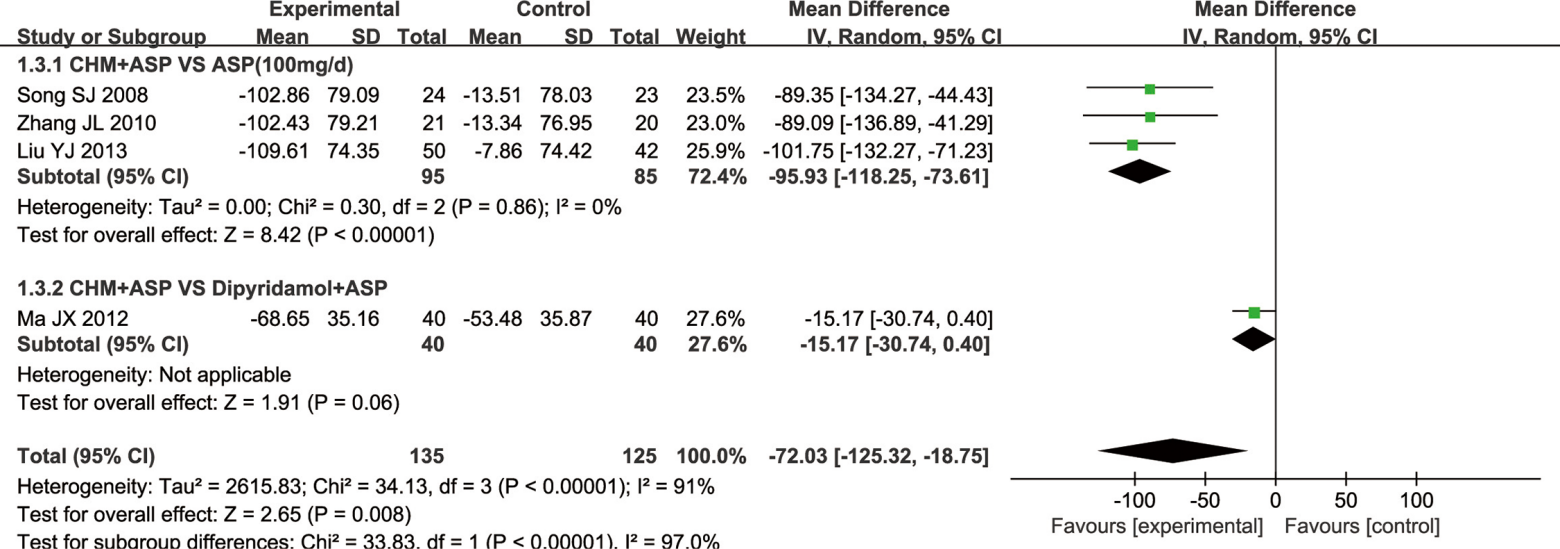
TXB_2_.

5 trials reported the TXB_2_, but only 4 of them could get the data and one (Su WJ 2012) didn’t report exact data. 3 trials adopted CHM plus aspirin versus regular dose aspirin, one adopted CHM plus aspirin versus high dose aspirin (300mg/d) and another one adopted CHM plus aspirin versus dipyridamole plus aspirin (Fig 8). All trials reported there was significant difference of decrease in TXB_2_ (Chi^2^ = 34.13, I^2^ = 91%, P<0.05) except Su WJ 2012.

**Fig 9:**
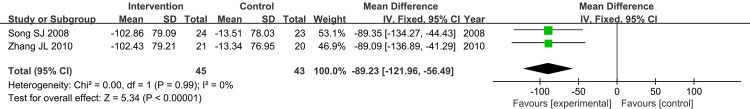
TXB2 (Tongxinluo Capsule +ASP vs ASP, ASP: Aspirin 100mg/d).

**3.1 Tongxinluo Capsule plus Aspirin versus Aspirin.** There were 2 RCTs that made a comparison of TXB2 between Tongxinluo capsule plus aspirin to aspirin (100mg/d). The meta-analysis showed there was a significant difference in TXB_2_ between Tongxinluo capsule plus aspirin versus aspirin (Fixed Effect model (FE), Standard Deviation (SD) = -89.23, 95% Confidential Interval (CI)[-121.96, -56.49], Chi^2^ = 0.00, I^2^ = 0%, P<0.00001) (Fig 9).

#### 4 Clinical Effective Rate

**Fig 10:**
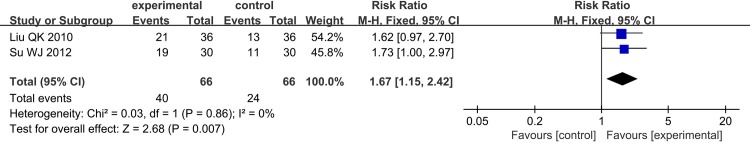
Clinical Effective Rate.

2 RCTs reported the clinical effective rate, and the meta-analysis result showed a significant difference in intervention and control group (FE, Relative Risk (RR) = 1.67, 95%CI[1.15, 2.42], Chi^2^ = 0.03, I^2^ = 0%, P = 0.007<0.05) (Fig 10).

#### 5 Cerebral Infarction Reoccurrence Rate

**Fig 11:**
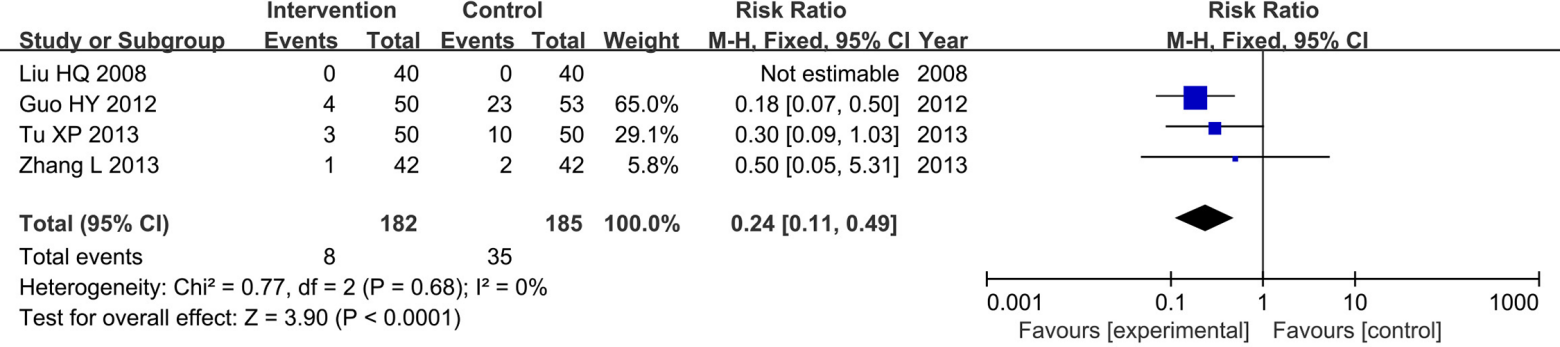
Cerebral Infarction Reoccurrence Rate.

Furthermore, cerebral infarction was observed in 4 trials, CHM plus aspirin had better effect of reducing the reoccurrence of cerebral infarction than aspirin (FE, RR = 0.24, 95%CI[0.11, 0.49], Chi^2^ = 0.77, I^2^ = 0%, P<0.0001) (Fig 11). And one trial (Huang HM 2014) showed that CHM plus aspirin could decrease the National Institutes of Health Stroke Scale (NHISS) score (P<0.05) and increase the Barthel Index (BI) score (P<0.05).

#### 6 Inflammation Indicator

Song SJ 2008 reported significant effect of Tongxinluo capsule plus aspirin for reducing C reactive protein (CRP) compared with aspirin (P<0.05). Liu YJ 2013 reported significant difference in the reduction of interleukin-6 (IL-6) by compared Sanqi Tongshu capsule plus aspirin with aspirin (P<0.05). Zhang X 2013 also showed significant effect in reducing the high-sensitivity C-reactive protein (hs-CRP).

#### 7 COL

The RCT (Yin CH 2010) using the electrical impedance to test the PAR induced by ADP and collagen (COL) showed significant differences in Tongxinluo capsule plus aspirin versus aspirin (P<0.05).

### Adverse Events

**Fig 12:**
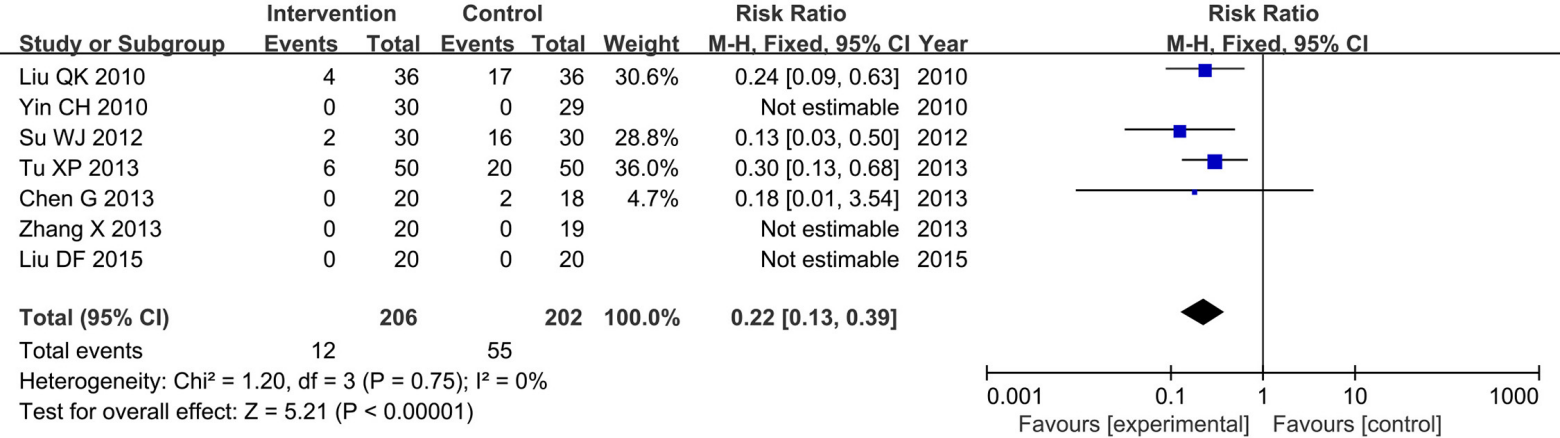
Adverse Events.

7 of the included 18 trials reported adverse events. Adverse events included stomach discomfort, dizziness, nausea, changes of blood pressure, an increase in clotting time and so on. Among 7 trials, 4 trials stated that there were no adverse events occurred in intervention group. There was significant difference of CHM or CHM plus aspirin in reducing the occurrence of adverse events (FE, RR = 0.22, 95%CI[0.13, 0.39], Chi^2^ = 1.20, I^2^ = 0%, P<0.00001, shown as Fig 12).

**Fig 13:**
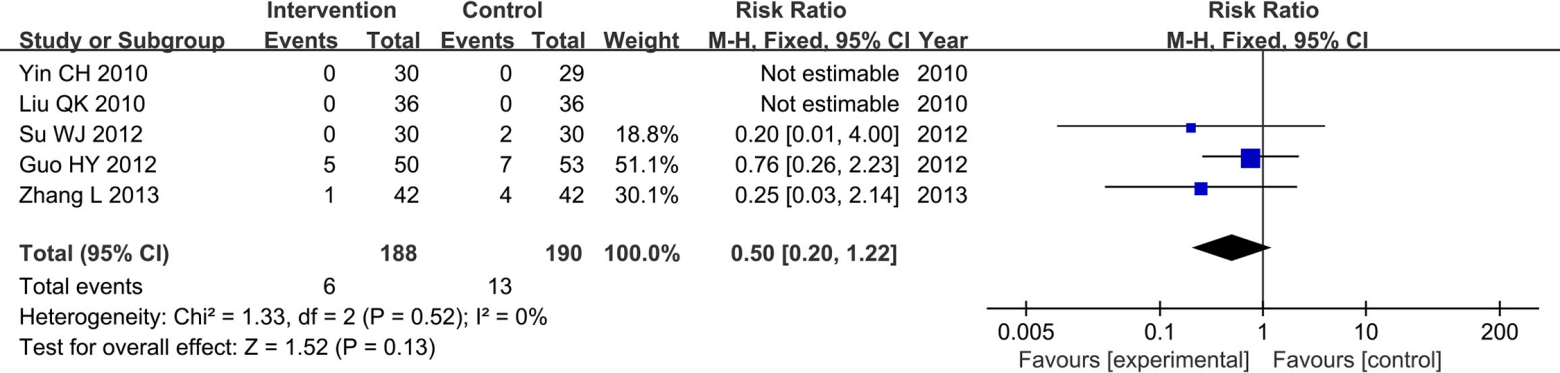
Bleeding Events.

Bleeding events were reported in 5 trials, and the rate of bleeding events of the intervention and control group had no significant difference. So the CHM monotherapy and CHM adjunctive therapy for AR would not add the risk of bleeding (FE, RR = 0.50, 95%CI[0.20, 1.22], Chi^2^ = 1.33, I^2^ = 0%, P = 0.13>0.05) (Fig 13).

## Discussion

### 1 Summary of Evidence

In this systematic review and meta-analysis of 1,460 patients from 18 studies of CHM for AR, we observed the results as following:(1) CHM could reduce the PAR induced by ADP and AA, TXB_2_ and could also reduce the cerebral infarction reoccurrence rate. (2) CHM monotherapy and CHM adjunctive therapy for AR would not add the risk of bleeding. (3) CHM plus aspirin could decrease the NHISS score (P<0.05) and increase the BI score (P<0.05). (4) There was significant difference in the reduction of C reactive protein (CRP) and interleukin-6 (IL-6) by comparing CHM plus aspirin with aspirin.

### 2 Significance of the Study

The phenomenon of AR also exits in China. More and more published researches in the past few years showed that the enthusiasm of applying CHM for AR is growing. Majority of CHM (88.9%) in our study was Chinese patent drug, which has a stringent quality control, and its quality is more stable than the water decoction of Chinese medicine.

2 RCTs applying Chinese patent drugs reported the clinical effective rate, and the meta-analysis result showed a significant difference in intervention and control group (RR = 1.67, 95%CI[1.15, 2.42], P = 0.86, I^2^ = 0%). This result has certain guiding significance for clinical application of Chinese patent drug for AR.

### 3 Application of Chinese Patent Drugs in the Therapy of AR

Tongxinluo capsule is a widely used Chinese patent drug for promoting blood circulation. Subgroup analysis showed that Tongxinluo capsule plus aspirin could be better at reducing PAR induced by ADP than aspirin. And one RCT reported significant effect of Tongxinluo capsule plus aspirin to reduce CRP. It would be used as alternative medicine of aspirin for secondary prevention of cardiovascular and cerebrovascular disease.

Compound danshen dripping pill is the patent drug which first successfully completed the United States Food and Drug Administration (FDA) phase II clinical trial. The phase II clinical trial results confirmed Compound danshen dripping pill’s effectiveness and safety. But the results of subgroup analysis in our study of the Compound danshen dripping pill showed a high clinical heterogeneity (Chi^2^ = 177.28, I^2^ = 99%, P<0.00001 and Chi^2^ = 20.27, I^2^ = 95%, P<0.00001). There may be several reasons: The course of treatment was not consistent (from 2 weeks to 1month). Patients with different kinds of diseases were included in these studies (Hypertension, Cerebral infarction and Coronary heart disease). Induction dose was also very different (ADP10umol/L or 1mmol/L, AA0.5mg/ml or 0.5mmol/L).

### 4 Adverse Events of CHM

Among 18 included studies, adverse events were reported in 7 studies. Adverse events included stomach discomfort, dizziness, nausea, changes of blood pressure, an increase in clotting time and so on. Among 7 trials, 4 trials stated that there were no adverse events occurred in intervention group. There was significant difference of CHM or CHM plus aspirin in reducing the occurrence of adverse events.

The results of subgroup analysis showed that CHM may be effective and safe as an alternative and collaborative therapy for AR. The CHM monotherapy (1 trial)and CHM adjunctive therapy(4 trials) for AR would not add the risk of bleeding events, meanwhile CHM plus aspirin had better effect of reducing the recurrence of cerebral infarction than aspirin. Although the sample size of some results were too small to draw very positive conclusions, the trends were very consentaneous.

### 5 Platelet Function Test Measures

The biochemical definition of AR is still controversial at present, the diagnostic criteria of AR are not unified. As we all know, platelet function plays an important role in the diagnostic criteria of AR. There are many kinds of methods to detect the function of platelet nowadays. Although studies have shown that the detection of platelet function in vitro is related to clinical events, there is not enough evidence to evaluate the clinical prognosis of aspirin-treated patients according to the results of platelet function test in vitro.

Platelet function is determined by COX-1 related pathway (arachidonic acid, AA) and other pathways (such as ADP or collagen induced platelet aggregation). While the specific detection method of AR should be the PAR induced by AA, such as light or optical transmission and the detection of Verify Now. Currently the FDA has approved the use of Verify Now detection method in clinical trials to observe the efficacy of aspirin for AR.

### 6 Related indicators of AR

TXB_2_, as the stable metabolite of TXA_2_, is an ideal indicator of adverse thrombotic events. The pooling data of meta-analysis showed the TXB_2_ in patients with CHM plus aspirin was significantly reduced. The CHM might reduce the PAR via keeping the balance of TXB_2_ and 6-keto-prostaglandin F1α(6-Keto-PGF1α).

TXA_2_ and prostacyclin (PGI_2_) are metabolites of AA. Under normal physiological conditions, TXA_2_ and PGI_2_ are in relative balance state to maintain the normal vascular tension and patency, and their balance disorders can cause vascular spasm and occlusion. TXB_2_ and 6-Keto-PGF1α are stable metabolites of TXA_2_ and PGI_2_. The contents of TXB_2_ and 6-Keto-PGF1α are determined by TXA_2_ and PGI_2_. The plasma TXB_2_and 6-Keto-PGF1α assay can reflect the overall activity of platelets, but have not been recommended for the diagnosis of AR at present. Whether TXB_2_ and 6-Keto-PGF1α can become new diagnostic indicators also depends on validation of more basic and clinical research.

A study, followed up 976 patients with high risk of cardiovascular events for 5 years, found that 11-dehydro thromboxane B_2_ (11-dTXB_2_) is an independent risk factor for cardiovascular events, having a high clinical value [38].

### 7 Aspirin Resistance and Inflammation

The results of subgroup analysis show significant difference in the reduction of CRP and IL-6by comparing CHM plus aspirin with aspirin (P<0.05). The latest study found that some inflammatory markers, such as CRP and IL-6 are associated with AR [39].

Aspirin only blocks COX-1 pathway of platelet activation and cannot block other platelet activation pathways, which may occur in patients with AR [40].

The decline in the level of inflammation markers has some inspiration for the further research. Ongoing trials of CHM therapy in patients with AR may provide more definitive conclusions. Results from upcoming large sample size trials would help clarify the effects of CHM with greater precision, including whether the benefits differ between male and female.

### 8 Mechanism of Aspirin Resistance in Chinese Medicine

The main patterns of syndrome distribution of patients with AR are Qi deficiency, phlegm dampness and blood stasis. Blood stasis syndrome accounts for a larger proportion. Qi deficiency and phlegm dampness syndrome are the pathological basis of AR. The etiology of blood stasis is not only the pathological basis of AR but also the pathological products of AR. Blood stasis is the cause of AR in the theory of Chinese medicine and plays a role in the accumulation of blood stasis and transformation for turbidity toxin. Blood stasis will finally develop into AR.

In TCM theory, constitution is closely related to the occurrence of diseases. And constitution determines the susceptibility to the disease and the tendency of the pathological changes. But the reported studies ignored the effect of constitution and syndrome differentiation. We only include the studies based on disease differentiation in order to affirm the clinical curative effect, and to reduce clinical heterogeneity.

In our opinion, TCM diagnosis and treatment system for AR should follow the model of differentiation of constitution, disease and syndrome. It is a clinical diagnostic and treatment model which reflects the nature, regulation, characteristics of the disease from different angles and different levels, and makeups for their limitations with each other.

### 9 Study Limitations

**Fig 14:**
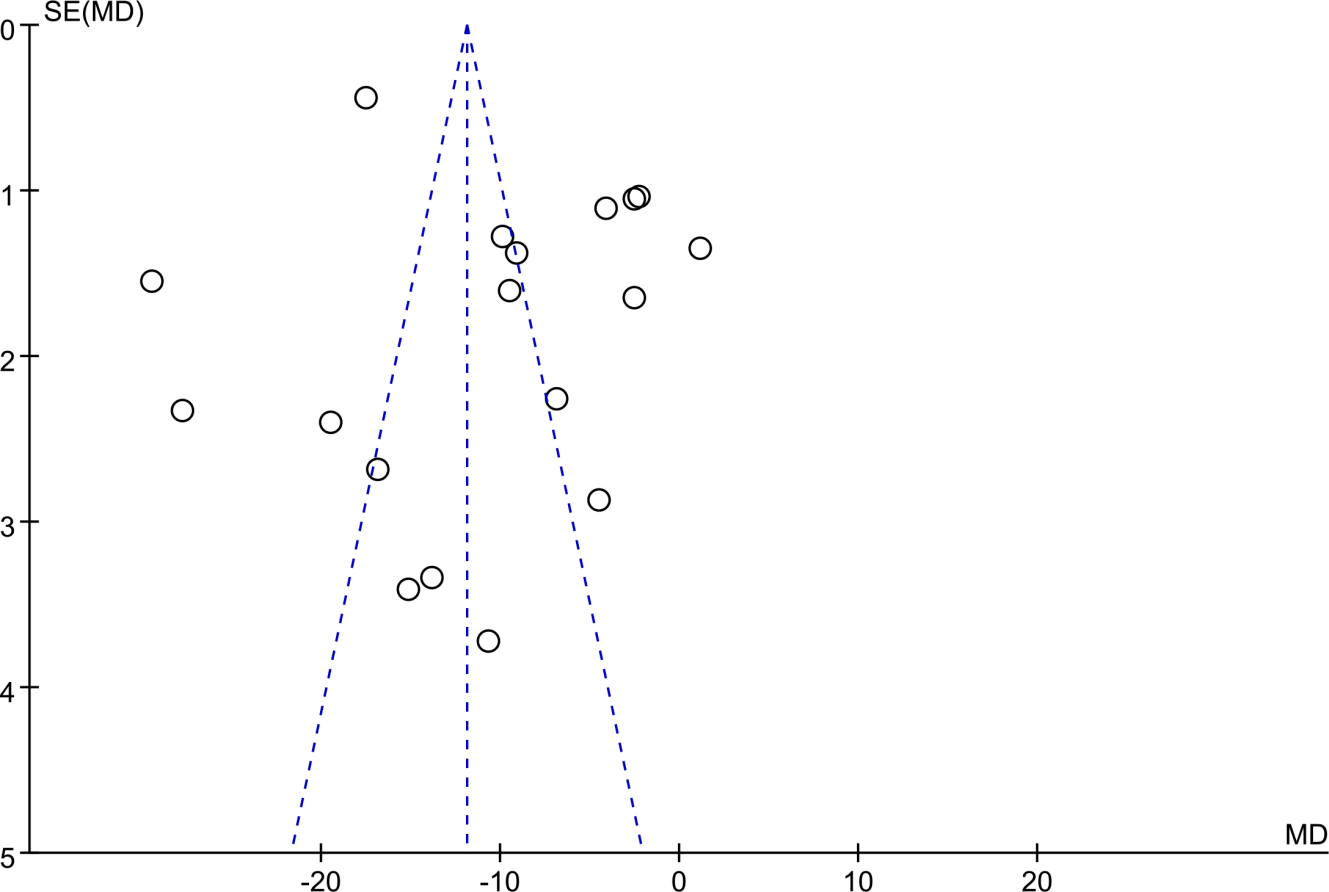
Funnel plot.

The study has several limitations that should be addressed:

1. Although the intervention and control groups were included in the study strictly, varied diagnostic criteria (such as different revulsants and induction dosages) may become an important factor for the clinical heterogeneity. The methodological quality of most included trials with small sample size was poor. Most trials had not been registered, and most of which did not mention random sequence generation, allocation concealment, blinding of participants and personnel, blinding of outcome assessments.
2. Great clinical heterogeneity existed in this systematic review and meta-analysis because different kinds of PAR testing measures, diagnosis criteria, and different CHM interventions are used in included trials involving various diseases.
3. Most of included trials conducted short term interventions (approximately four weeks, only 2 studies up to 12 weeks) which may not be long enough to reflect the long-term effect of CHM for AR.
4. We performed a funnel plot of 18 trials involved PAR induced by ADP to analyze the public bias. The funnel plot implied that the public bias existed in these trials (Fig 14).

### 10 Implications for Future Directions

Although this study shows that CHM may be effective and safe for AR treatment as a kind of complementary and alternative medical therapy, whether CHM is used alone or in combination with aspirin still need to be tackled:

The current evidence and potential findings should be interpreted carefully because of poor methodological quality of these studies, insufficient evidence of effectivity and safety and the clinical heterogeneity. Further rigorous RCTs are called to overcome the limitations.

Contemporary, we should pay attention to the problems that why particular patients do not benefit from aspirin therapy and how to identify them? Since the mechanisms of AR still require further research and specific therapy remains absent, new studies need to be designed to sift out the most appropriate test measures to diagnosis AR and to find out economical alternative therapies that are effective for AR patients. The diagnosis criteria of AR should be unified.

Large scale clinical research should be carried out to compare the reliability and sensitivity of different detection in AR diagnosis, and to observe the incidence of cardiovascular events in varied treatment carefully.

So far, western medicine has not yet formed a comprehensive prevention and treatment strategy of AR, the treatment of TCM syndrome differentiation and treatment has a promising application prospect in the therapy of AR.

The treatment integrating Chinese and Western medicine for AR should be based on the dual diagnosis. Combining western medicine with the treatment by differentiation of constitution, disease and syndrome in TCM theory is better for AR therapy. But no unified diagnostic criteria for AR in TCM theory at present brings some difficulties for the dual diagnosis.

## Conclusions

Our study is a new systematic review and meta-analysis of CHM for AR, which is also the first meta-analysis on clinical outcomes, clinical effective rate and adverse events of CHM for AR. It suggests that CHM may be effective and safe for the treatment of AR, and can be used as an alternative medicine of aspirin for enhancing pharmacy efficiency, decreasing the risk of hemorrhage and reoccurrence of cerebral infarction. However, owing to poor methodological quality of included studies, different diagnosis criteria and determination methods, and the varied CHM interventions, the current evidence and potential promising findings should be interpreted with caution. In the future, rigorous multicenter, large sample size clinical RCTs are proposed to surmount the limitations of current trials to improve the strength of evidence. And the diagnosis criteria of AR should be unified.

## Supporting Information

S1 Checklist PRISMA Checklist of CHM for AR SR. (PDF)

S1 Text CRD of CHM for AR SR. (PDF)
