# Supplementary material for: Chinese Herbal Medicine for Aspirin Resistance: A Systematic Review and Meta-Analysis
Source: PLoS One. 2016 May 6;11(5):e0154897. doi: 10.1371/journal.pone.0154897 (PMC4859478; doi:10.1371/journal.pone.0154897)
Supplement: S1 Text — (PDF) [file pone.0154897.s002.pdf]

PROSPERO International prospective register of systematic reviews

Chinese herbal medicines for aspirin resistance: a systematic review for randomized controlled trials

Hanyu Chen, Zhengjie Shen, Haowen Zhang, Jiandong Chen, Xiaohu Chen

Citation

Hanyu Chen, Zhengjie Shen, Haowen Zhang, Jiandong Chen, Xiaohu Chen. Chinese herbal medicines for aspirin resistance: a systematic review for randomized controlled trials. PROSPERO 2015:CRD42015020182 Available from http://www.crd.york.ac.uk/PROSPERO_REBRANDING/display_record.asp?ID=CRD42015020182

Review question(s)

To evaluate the efficacy and safety of traditional chinease medicine used for therapy of AR

Searches

English database: MEDLINE (OVID SP), EMBASE (OVID SP), Cochrane Central Register of Controlled Trials (CENTRAL).

Chinese database: the Chinese BioMedical Database(CBMDisc),CMCI, Chinese National Knowledge Infrastructure Database(CNKI), VIP information database, and Wanfang Data Information Site

Types of study to be included

We included randomized controlled trials (RTC) published home and abroad concerned with using TCM to treat AR, with no restrictions on particular treatment, whether carry out blinding or allocation concealment, publication status. Language is restricted to Chinese and English. But quasi-randomised trials carried out according to their date of birth, the date to which the patients were sent to the hospital, ID number,their hospital record number, will be excluded.

Condition or domain being studied

Aspirin resistance has been a prominent clinical problem in recent years. Cardiovascular diseases cannot be effectively prevented and controlled if AR exists, repeatedly attacking and increasing the mortality, have induced some social burdens. Now Western medicine is still at the stage of discovery on treating this problem and it lacks specific treatment. While traditional Chinese medicine is also carrying out clinical research on AR, and some related scholars have done a systematic assessment on the clinical research done before 2012 about how TCM interfere AR. But they did not get certain conclusions due to the bad quality of the inclusive clinical research. It has been two years since the old systematic review, when some new clinical researches about it have been reported. We aim to do a new systematic review by accumulating the existed evidence so as to evaluate and observe the efficacy and safety of TCM on interfering AR, providing new treatment for antiplatelet-aggregation, which makes important sense.

Participants/ population

We will include participants of any age or sex who were diagnosed with AR.

Diagnostic criteria:

1. 10μmol/L adenosine diphosphate (ADP)-induced platelet aggregation rate =70%

2. 0.5mmol/L arachidonic acid(AA)-induced platelet aggregation rate=20%.

It can be diagnosed as AR if it meets both 1 and 2. The diagnostic criterion of aspirin semi-resistance (ASR) is meeting one of the two conditions above.

Exclusion criteria: aspirin allergy; cerebral hemorrhage or bleeding tendency; active gastric ulcer and gastrointestinal bleeding patient; liver damage; all kinds of blood disease; abnormal platelet count; aspirin allergy; Pregnancy or

breast-feeding women; using drugs effecting platelet aggregation during the last 1 month of observation period.

Intervention(s), exposure(s)

The Chinese herbal medicine(CHM) interventions included single herbs, extracts from any single herb, Chinese patent drug (for example oral liquid, capsule, tablet, pill, powder, or injection), or traditional Chinese prescriptions prescribed by TCM doctors.

Therapy in the treatment groups: any treatments using CHM monotherapy or adjunct therapy for AR was included regardless of the dose, formulations and frequency of CHM.

The treatment course was at least 2 weeks in these trials included.

Comparator(s)/ control

Interventions of the control groups: aspirin (regardless of the dose), placebo , no intervention, or other conventional antiplatelet drug treatment. The treatment course was at least 2 weeks in these trials included.

Outcome(s)

Primary outcomes

The variation of the platelet aggregation rate induced by AA/ADP, TXB2 and 6-KETO-PGF1a.

We will measure the outcome in the beginning and at the end of the treatment course.

Secondary outcomes

Clinical efficacy and safety.

Standard of the clinical efficacy:

1. AR becomes ASR or aspirin sensitive after the treatment;

2. ASR becomes aspirin sensitive after the treatment;

3. the decreased value of platelet aggregation rate is more than 10% and platelet active value is normal.

Risk of bias (quality) assessment

We will evaluate included studies for risk of bias using a tool for assessing risk of bias of RTC introduced in the Cochrane Handbook for Systematic Reviews of Interventions (V.5.1.0). In particular, we studied the following six domains:

1. random sequence generation

2. Allocation concealment

3. blinding of participants, personnel and outcome assessors;

4. completeness of outcome data, including baseline measured before intervention, effects parameter after intervention, dropouts and withdrawals (whether the loses visit rate is under 10%), excluded data, whether or not they explained the reason of losses to follow-up, whether or not ITT Analysis is carried out in people lost to follow-up;

5. selective outcome reporting: Whether or not safety problems (e.g. adverse events such as death) and negative results are reported

6. other sources of bias: early termination of trials, imbalance baseline?

For each included article, make judgment of “yes” (low risk of bias), “no” (high risk of bias) or “unclear” (indicating unknown or unclear risk of bias) about the above mentioned six domains.

Strategy for data synthesis

We planned to carry out the meta-analyses in Revman software. We will use a fixed-effect model for meta-analysis if there is no statistical heterogeneity between primary and secondary outcomes measures (P >0.1,I-squared = 50%); If we had identified clinical homogeneity but statistical heterogeneity (P<0.1,I-squared >50%), the random-effects model would have been used.

Heterogeneity will be assessed using both the Chi-squared test and the I-squared statistic. We will consider an I-squared value greater than 50% to be indicative of substantial heterogeneity. We will conduct sensitivity analyses based on study quality, if necessary. Funnel plots will be generated to detecting publication bias if more than ten trials are identified.

Analysis of subgroups or subsets

If the necessary data are available, subgroup analyses will be done for different outcome measures.

Contact details for further information

Dr Chen

Nanjing University of Chinese Medicine, 138 Xianlin Rd., Nanjing 210046, P.R.C

chenhanyu@163.com

Organisational affiliation of the review

1.Nanjing University of Chinese Medicine 2.Jiangsu Province Hospital of TCM Affiliated Hospital of Nanjing University of TCM

1.www.njucm.edu.cn 2.www.jshtcm.com

Review team

Dr Hanyu Chen, Nanjing University of Chinese medicine
Dr Zhengjie Shen, Nanjing University of Chinese Medicine
Dr Haowen Zhang, Nanjing University of Chinese Medicine
Dr Jiandong Chen, Jiangsu Province Hospital of TCM Affiliated Hospital of Nanjing University of TCM
Professor Xiaohu Chen, Jiangsu Province Hospital of TCM Affiliated Hospital of Nanjing University of TCM

Anticipated or actual start date

03 March 2015

Anticipated completion date

03 August 2015

Funding sources/sponsors

National Natural Science Foundation of China NO.81273943

Conflicts of interest

None known

Country

China

Subject index terms status

Subject indexing assigned by CRD

Subject index terms

Aspirin; Complementary Therapies; Drugs, Chinese Herbal; Humans; Phytotherapy

Stage of review
Ongoing

Date of registration in PROSPERO
01 May 2015

Date of publication of this revision
04 August 2015

Stage of review at time of this submission	Started	Completed
Preliminary searches	Yes	Yes
Piloting of the study selection process	Yes	Yes
Formal screening of search results against eligibility criteria	Yes	No
Data extraction	No	No
Risk of bias (quality) assessment	No	No
Data analysis	No	No

PROSPERO
International prospective register of systematic reviews
The information in this record has been provided by the named contact for this review. CRD has accepted this information in good faith and registered the review in PROSPERO. CRD bears no responsibility or liability for the content of this registration record, any associated files or external websites.
